# Phytohormones and emerging plant growth regulators in tailoring plant immunity against viral infections

**DOI:** 10.1111/ppl.70171

**Published:** 2025-03-24

**Authors:** Kritika Shukla, Nikita, Altaf Ahmad, Md Salik Noorani, Ravi Gupta

**Affiliations:** ^1^ Plant Molecular Virology Lab, Department of Botany, School of Chemical and Life Sciences Jamia Hamdard New Delhi India; ^2^ Department of Botany, Faculty of Life Sciences Aligarh Muslim University Aligarh Uttar Pradesh India; ^3^ Plant Stress Physiology and Proteomics Laboratory, College of General Education Kookmin University Seoul South Korea

## Abstract

Viral infections are major contributors to crop yield loss and represent a significant threat to sustainable agriculture. Plants respond to virus attacks by activating sophisticated signalling cascades that initiate multiple defence mechanisms. Notably, several phytohormones, including salicylic acid (SA), jasmonic acid (JA), abscisic acid (ABA), and ethylene (ET), are known to shape these defence responses. In recent years, various plant growth regulators (PGRs) such as melatonin, carrageenans, sulfated fucan oligosaccharides, nitric oxide (NO), brassinosteroids (BRs), and hydrogen sulfide (H_2_S) have also emerged as crucial regulators of plant defence responses against virus infections. Emerging evidence indicates that these PGRs coordinate with phytohormones to activate various defence strategies, including (1) stomatal closure to limit pathogen entry, (2) callose deposition to block plasmodesmata and restrict viral spread within host tissues, (3) attenuation of viral replication, and (4) activation of RNA interference (RNAi), a crucial antiviral defence response. However, the interactions and crosstalk between PGRs and phytohormones remain largely underexplored, thereby limiting our ability to develop innovative strategies for managing viral diseases. This review discusses the diverse functions and crosstalk among various phytohormones and PGRs in orchestrating the plant defence mechanisms, highlighting their impact on viral replication, movement, and intercellular transport.

## 1 INTRODUCTION

**FIGURE 1:**
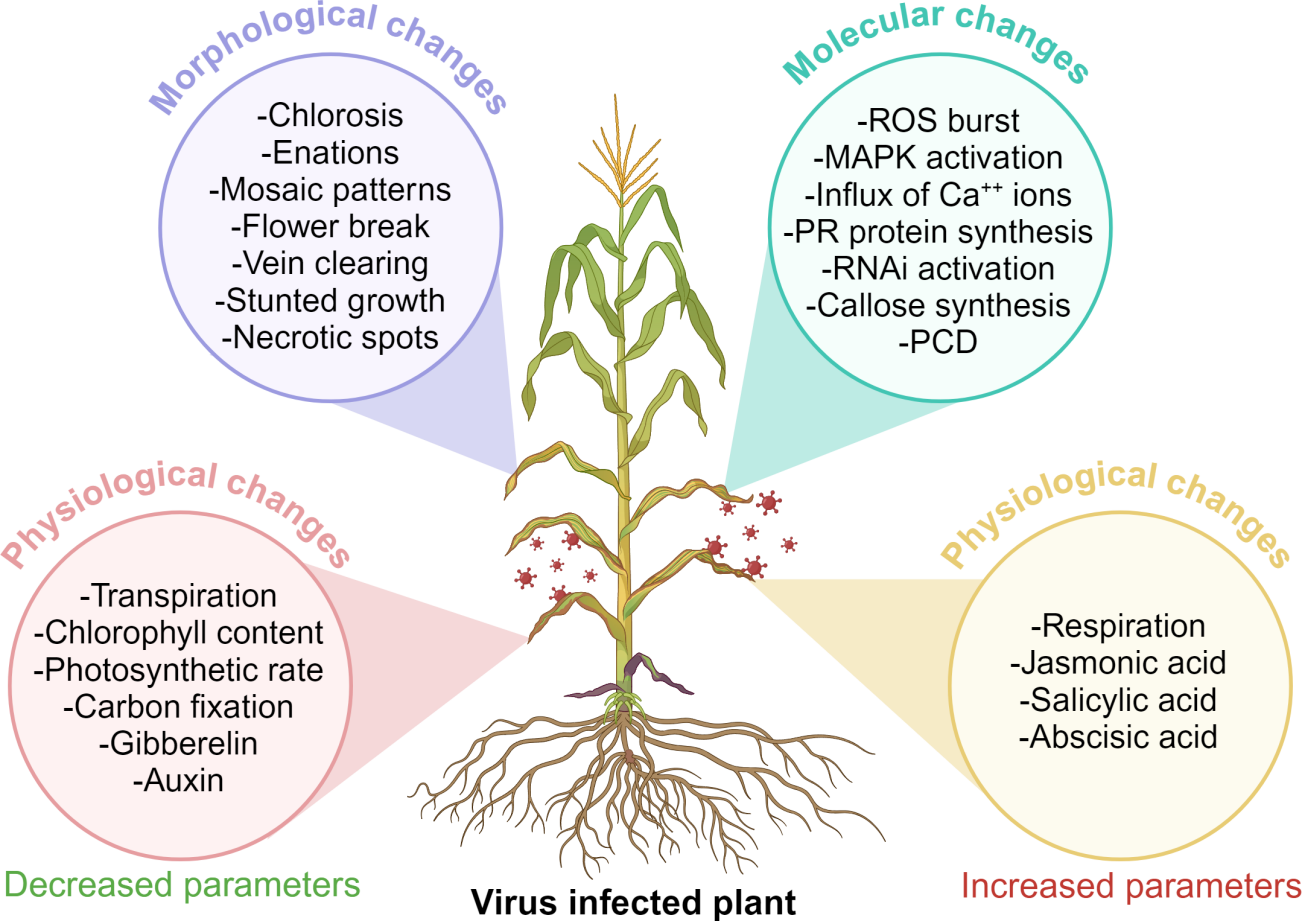
**Morphological, physiological, and molecular changes in plants under viral infection**. Figure created with BioRender.

Viruses pose a significant threat to crops, causing damage and adverse effects on agronomical traits such as grain quality and yield (Gupta et al., 2018). The annual damage imposed by viruses has been estimated to be 30 billion US dollars globally (Sastry & Zitter, 2014). Environmental conditions, along with anthropogenic factors such as changes in agricultural practices and global trade, are known to contribute to the emergence of new viral diseases (Jones & Naidu, 2019). Tobacco mosaic virus (TMV), cauliflower mosaic virus (CaMV), cucumber mosaic virus (CuMV), and rice black‐streaked dwarf virus (RBSDV) are some of the most common and highly contagious viruses that infect various crops and are responsible for the majority of the virus‐induced cross loss (Sastry & Zitter, 2014; Singhal et al., 2021). At the morphological level, the virus infections can be evidently monitored by the presence of chlorotic lesions, ring spots, mosaic patterns, vein clearing, and necrotic spots on stems, leaves, and fruits, among others (Figure 1). The chlorosis symptoms linked to viral infections are likely caused by disruption of chloroplast components and functions (Manfre et al., 2010). Notably, these virus infections induced changes in chloroplast structure and functions result in reduced photosynthetic activity which is a typical cause of altered plant growth and productivity (Zhao et al., 2016). At the molecular level, virus‐secreted RNA/proteins interact with the host proteins to manipulate their functions, leading to the hijacking of host cellular machinery. Viral RNA/proteins also manipulate key signalling components to suppress the host immune response. Host cells, in turn, retaliate by triggering the biosynthesis of defence proteins, accumulation of callose in plasmodesmata, and sometimes activation of the RNA interference (RNAi) pathway (Figure 1) (Incarbone & Dunoyer, 2013). Intriguingly, the majority of these cellular events are commenced as a result of activation of one or more phytohormones signalling that not only fine‐tunes the cellular response but also shapes the plant immune response depending on the specific virus (Figure 1).

Phytohormones such as salicylic acid (SA), jasmonic acid (JA), abscisic acid (ABA), and ethylene (ET) are closely associated with the activation of plant defence response (Gupta et al., 2024). Among these, SA is known to induce immune responses against viruses, including R‐gene‐mediated resistance, systemic acquired resistance (SAR), and basal defence processes. Notably, crosstalk between SA and JA/ET pathways is often observed, which regulates defence against different viral pathogens (Koornneef & Pieterse, 2008; Takahashi et al., 2004). In addition, ABA has been shown to limit viral movement by enhancing callose deposition (Iriti & Faoro, 2008), while JA is associated with resistance against the geminivirus infection (Lozano‐Durán et al., 2011). Along with these classical phytohormones, some of the emerging plant growth regulators (PGRs) are also turning out to be crucial regulators of plant defence responses. PGRs include natural and synthetic compounds that exhibit regulatory roles in the growth and development of plants during normal and challenging environmental conditions. Additionally, recent research has also highlighted the roles of some of the relatively uncharacterized components, such as carrageenans, in improving plant resistance against viral infections (Ries et al., 1977; Sangha et al., 2015; Sofy et al., 2020; Moskova et al., 2021).

In this review, we are presenting the intricate roles of diverse phytohormones and emerging PGRs in fortifying plant immunity against viral infections. The insights gleaned from this review can potentially contribute to the development of innovative strategies for effective management and control of viral diseases in crop plants, ultimately safeguarding global food security and sustainable agriculture.

### 1.1 Plant defence response: An overview

Plants have developed an intricate defence system to identify pathogens and accordingly trigger local and systemic responses. Plants deploy a variety of defence strategies, such as RNAi, protein degradation, and metabolic regulation, to prevent virus replication and spread inside the plant tissues (Incarbone & Dunoyer, 2013). The first layered immune response, the so‐called P/MAMP‐triggered immunity (PTI), is activated when certain conserved pathogen‐ or microbe‐associated molecular patterns (P/MAMPs) are recognized by surface‐localized plant pattern recognition receptors (PRRs) (Boller & Felix, 2009; Dodds & Rathjen, 2010; Schwessinger & Ronald, 2012, Gupta et al., 2015). Apart from PAMPs, the PTI system may also identify plant endogenous peptide (Pep) elicitors, which are believed to enhance the defence reactions against invasive microbes (Yamaguchi & Huffaker, 2011). Damage‐associated molecular patterns (DAMPs) are a collective term for Peps and other endogenous elicitors, released upon cell damage, that trigger a defence response in plants (Boller & Felix, 2009). It is plausible that viral infections for which no conserved PAMP has been identified so far may elicit PTI‐based responses through the activation of endogenous DAMPs (Ding & Voinnet, 2007). Since viruses are exclusively intracellular pathogens, Viral PAMPs (VAMPs), like DAMPs, are released in the apoplast either actively or in response to cellular injury (Leonetti et al., 2021). When VAMPs, typically double‐stranded (ds)RNA of viral origin, are recognized by Dicer‐like proteins (DCLs) or transmembrane leucine rich PRRs, they can either trigger RNAi‐based antiviral defence or a classical PTI, respectively.

Minutes after P/DAMP recognition, signalling cascades set off a series of pathogen‐related reactions, including increased intracellular Ca^2+^ levels, generation of reactive oxygen species (ROS), activation of multiple kinases such as calcium‐dependent CDKs and mitogen‐activated protein kinases (MAPKs), leading to subsequent phosphorylation of downstream signalling proteins, and alterations in gene expression (Figure 1) (Dodds & Rathjen, 2010; Torres, 2010; Tena et al., 2011). However, in order to establish a robust infection, host‐adapted pathogens are able to interfere and overcome PTI by deploying effector proteins (virulence factors) directly into the host cytoplasm (Díaz‐Pendón & Ding, 2008; Teixeira et al., 2019). A second line of defence, termed effector‐triggered immunity (ETI), is activated following the identification of these intracellular pathogen effector proteins by the intracellular immune receptors. These intracellular receptors are the products of R‐genes that possess nucleotide‐binding leucine‐rich repeat (NB‐LRR) domains to identify viral effectors (Díaz‐Pendón & Ding, 2008; Teixeira et al., 2019). Tobacco *N* (TIR‐NB‐LRR), potato *Rx1* (CC‐NB‐LRR), and *Arabidopsis HRT* (CC‐NB‐LRR) are the most researched R‐genes for RNA viruses that provide resistance to tobacco mosaic virus (TMV), potato virus X (PVX), and turnip crinkle virus (TCV), respectively (Zvereva & Pooggin, 2012). ETI is associated with the recognition of pathogens or pathogen isolates with much greater specificity and triggers a considerably more robust defence response than PTI, frequently culminating in HR, a programmed cell death (PCD) to kill the pathogen‐infected cells (Mur et al., 2008). HR is a tightly controlled process and is mediated by several genetic, molecular, and biochemical events. In particular, the role of MAP kinases has been highlighted during the onset of HR, which subsequently triggers the expression of numerous defence‐related proteins, such as glucanases, chitinases, and defensins, among various others (Mur et al., 2008). Many of these defence‐related proteins are also known as pathogenesis‐related (PR) proteins and are produced concurrently with HR induction. Most plant PR proteins share common biochemical properties, *i.e*., they are low molecular weight, acid soluble, and protease resistant (Leubner‐Metzger & Meins Jr., 1999). Plants produce and accumulate PR genes in response to pathogen attacks, aiding in limiting virus spread, replication, and symptom development (Leubner‐Metzger & Meins Jr., 1999). In addition to locally accumulating in the infected and surrounding tissues, the PR proteins also accumulate in distant uninfected regions, protecting the plants from more infection, and thus, it is often associated with systemic acquired resistance (SAR) or HR against viruses. Additionally, some host proteins, like lectin proteins, also contribute to viral resistance responses. To distinguish between self‐ and non‐self‐originated carbohydrates, lectins in plants attach to mono‐ or oligosaccharide molecules, making them perfect for pathogen recognition (Van Damme et al., 2004). For example, an *Arabidopsis* jacalin‐type lectin, RESTRICTED TEV MOVEMENT1 (RTM1), mediates resistance against the tobacco etch virus (TEV) (Chisholm et al., 2000). It has been found recently that JAX1, another jacalin‐type lectin, confers resistance against several potexviruses, including Asparagus virus 3, Plantago asiatica mosaic virus (PLAMV), PVX, and White clover mosaic virus 4 (Yamaji et al., 2012). Comparing lectin‐mediated resistance (LMR) to NB‐LRR resistance reveals an interesting feature: LMR does not trigger HR and SAR responses, nor does it modify SA levels, signalling, or other common defensive gene expression alterations that are frequently altered in immunological resistance responses (Yamaji et al., 2012). The suggested working model of LMR activity (Yamaji et al., 2012; Chisholm et al., 2000) states that resistance is triggered by lectin proteins like JAX1 and RTM1 recognizing certain viral proteins that are glycosylated within plant cells. RTM1 may prevent viral movement by interfering with viral movement‐associated proteins, whereas LMR responses may prevent viral replication in the case of JAX1 by promoting the aggregation of replicase‐associated proteins. Another study identified a class of endoplasmic reticulum (ER)‐residing chaperones that affect antiviral immune responses using complementary proteomic approaches (Caplan et al., 2009). Within two hours following TMV infection, plants expressing the *N*‐gene in *N*. *benthamiana* exhibit significant expression of the ER proteins ERp57, P5, calreticulin2, and CRT3 (Caplan et al., 2009). N‐mediated resistance against TMV is lost as a result of virus‐induced gene silencing (VIGS) of the ER chaperones; however, VIGS only partially eliminates the symptoms of cell death and necrosis as well as TMV movement in the upper non‐inoculated leaves, most likely because it did not completely knock down the respective transcripts (Mandadi & Scholthof, 2012). SAR, yet another level of defence response in plants, may also be activated in distant uninfected tissues in response to an infection downstream of SA. However, SAR, in contrast to HR, is a sustained immune response that offers activation of immune responses in distant tissues against future infections (Roychowdhury et al., 2024). Notably, both of these processes are mediated by H_2_O_2_, which exhibits a two‐phase accumulation in response to virus attacks. While the early phase H_2_O_2_ burst is associated with the activation of defence signalling, which triggers the SA biosynthesis, expression of defence genes, callose deposition, and SAR induction, the second phase H_2_O_2_ burst is involved in the execution of HR. In addition to the H_2_O_2_, some other compounds such as glycerol‐3‐phosphate, N‐hydroxypipecolic acid, pipecolic acid, lipid transfer protein DIR1, azelaic acid, dehydroabietinal, methyl salicylate and extracellular NAD(p)/eNAD(p) are also known to be associated with SAR as either their accumulation has been observed during SAR induction or their exogenous treatment induces SAR (Gupta, 2025). Intriguingly, a recent study revealed that H_2_O_2_ is the mobile signal for the SAR, which governs SA biosynthesis in the systemic tissues. H_2_O_2_ produced during pathogen attack by the activity of NADPH oxidases in the local tissues is translocated to the systemic tissues to sulfenylate the CCA1 HIKING EXPEDITION (CHE) transcription factor to shape the SA biosynthesis during induction of SAR (Cao et al., 2024). Notably, this observation was recorded in Arabidopsis only in response to bacterial pathogens and therefore, the role of H_2_O_2_ as a mobile signal for SAR induction in response to viral pathogens still needs to be investigated.

Viral infections can also result in the simultaneous induction of several phytohormones that elicit the defence responses in the host plants (Alazem & Lin, 2015). Accordingly, changes in the levels of defence hormones such as SA, JA, and nitric oxide (NO), along with the accumulation of ROS, have been reported in multiple plants in response to viral infections (Sharma & Prasad, 2024). At the cellular level, alterations in membrane potential and permeability, changes in calcium (Ca^2+^) ion concentrations, and callose depositions over cell walls have been reported in the host cells (Mur et al., 2008). However, the facts that (a) the majority of plant viruses contain RNA genomes, (b) no conserved PAMP has been identified so far from plant viruses, and (c) the absence of evidence that immune receptors in plants can recognize viral RNA or DNA, suggests that the plant immune responses to viral pathogens are relatively different as compared to the defence responses activated in response to other pathogen types. Therefore, in addition to the PTI and ETI defence responses, plants also deploy an evolutionary conserved, unique kind of adaptive gene regulation known as RNA silencing or RNAi, which is triggered by parasitic double‐stranded (ds) RNA in a highly sequence‐specific manner (Ding & Voinnet, 2007). The RNAi system has evolved to recognize and target viral nucleic acids and is considered a primary defence response against viruses (Zvereva & Pooggin, 2012). RNAi system requires the sequential functioning of various proteins, including Dicer or Dicer‐like (DCL) enzymes, double‐stranded RNA binding (DRB) proteins, argonaute (AGO), RNA‐dependent RNA polymerase (RDR), and various accessory proteins. The *Arabidopsis thaliana* genome contains four DCLs (DCL1‐4), five DRBs (DRB 1–5), ten AGOs, and six RDRs that are specialized for several silencing‐related processes (Vaucheret, 2006).

**FIGURE 2:**
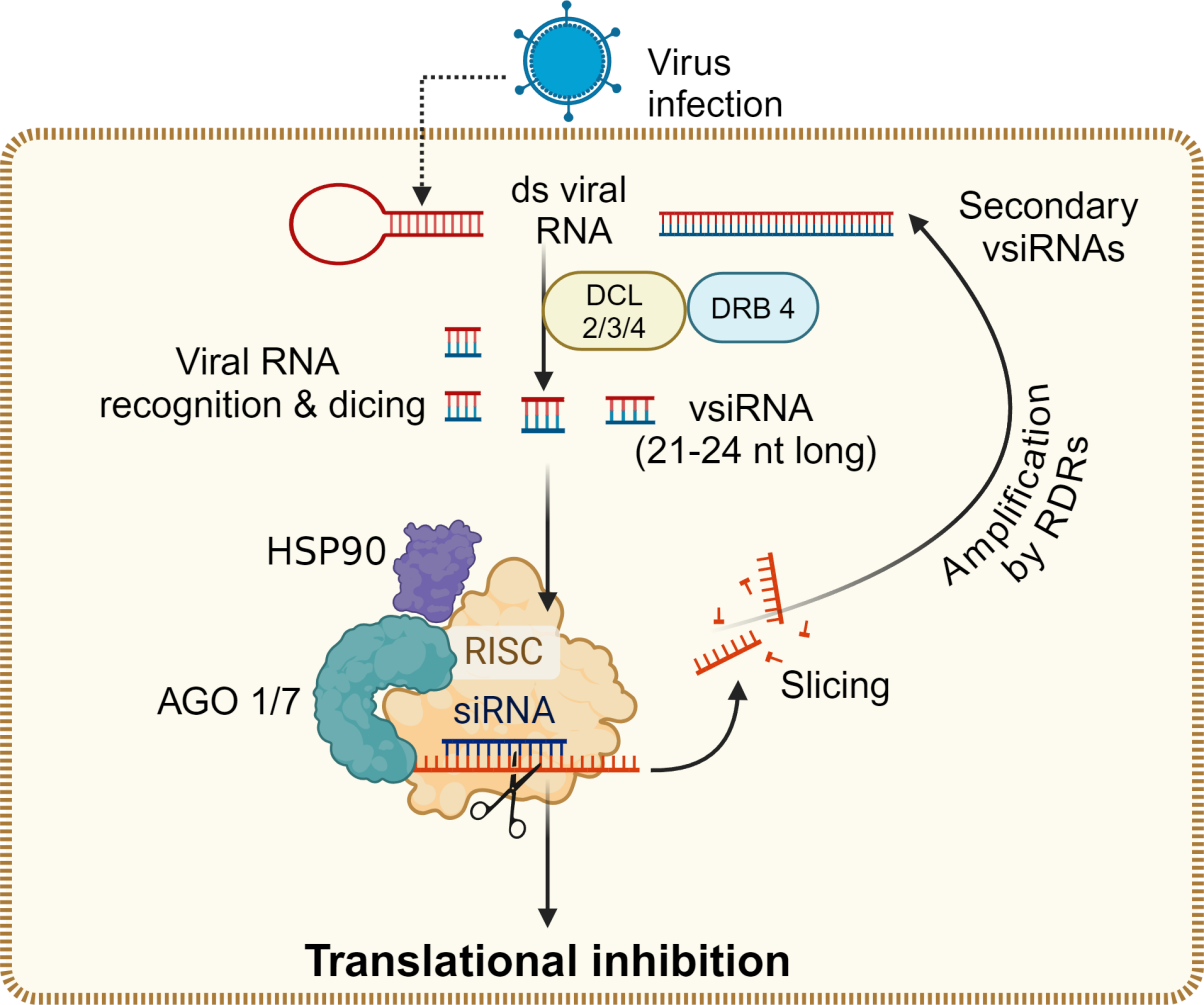
**Activation of RNA silencing in plant cells in response to viruses**. Plants can recognize the viral double‐stranded RNAs (dsRNAs) or partially ds hairpin RNAs to activate RNA silencing, leading to the formation of Virus‐derived siRNA (vsiRNA) by the activity of dsRNA‐specific RNases called Dicer or Dicer‐like (DCL) enzymes (DCL2/3/4) and its cofactor double‐stranded RNA binding (DRB) proteins (DRB4). HSP90‐activated argonaute (AGO) protein (AGO1/7) then binds to the vsiRNA to form an RNA‐induced silencing complex (RISC). RISC/AGO/vsiRNA then targets and degrades complementary viral transcripts to inhibit their translation. RNA‐dependent RNA polymerase (RDR) enzymes are associated with the formation of secondary vsiRNAs. Figure created with BioRender.

Plant DCLs are members of the RNase III type enzymes that transform the dsRNA precursors into 21–24 nt small (s)RNAs, which are then responsible for silencing (Figure 2). DCLs contain a helicase, two RNase‐III, a PAZ, and two dsRNA‐binding domains. DRB proteins collaborate with DCLs for the precise and efficient excision of sRNAs from their precursor molecules by functioning as DCL cofactors. sRNAs, formed because of the activity of DCL enzymes, bind to AGO proteins, which direct the formation of RNA‐induced silencing complex (RISC) in order to silence complementary DNA or RNA. AGO proteins are typically classified into three groups: true AGOs (Plant AGOs), WAGO proteins, and PIWI proteins, which perform a variety of roles that partially overlap. AGO conformational changes are driven by the Heat shock protein (Hsp) 70‐Hsp90 chaperone machinery and ATP hydrolysis during the AGO‐loading process. The RDR proteins are also required for both cellular and antiviral siRNA biogenesis (Figure 2). Virus‐derived siRNA (vsiRNA) in plants have been categorized as primary and secondary siRNAs. While primary siRNAs are produced when an initial trigger RNA is cleaved by DCL, secondary siRNAs are biosynthesized in the presence of an RDR enzyme (Ruiz‐Ferrer & Voinnet, 2009).

**FIGURE 3:**
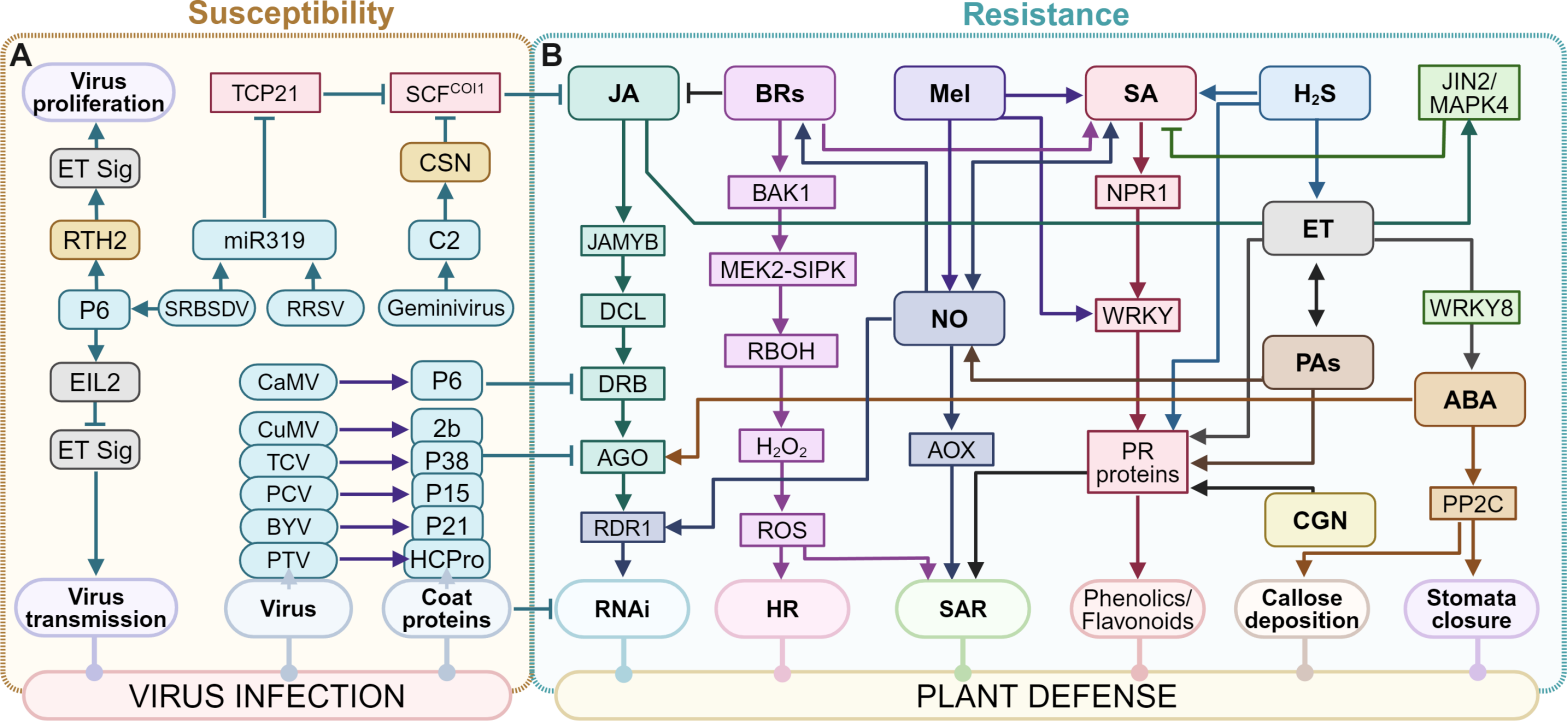
**Molecular mechanism underlying phytohormones mediated regulation of plant‐virus interactions**. (**A**) Viruses suppress plant immunity to promote their infection by inhibiting one or more components of plant immune responses. The C2 protein of Geminivirus suppresses the jasmonic acid (JA) response to viral infection by binding to CSN (COP9 signalosome) and influencing SCF (Skp, Cullin, F‐box containing complex) activity. Rice ragged stunt virus (RRSV) and S/RBSDV (Southern/rice black‐streaked dwarf virus) express miR319 that downregulate TEOSINTE BRANCHED1‐CYCLOIDEA‐PROLIFERATING CELL FACTOR (TCP21) to attenuate JA‐mediated defence. In addition, coat proteins from different viruses interact with different components of RNA‐induced silencing complex (RISC) to suppress RNA interference (RNAi), a key defence mechanism against viruses. Infection with RRSV and SRBSDV causes the production of miR319, which decreases TCP21's function and suppresses JA‐mediated resistance to promote virus infection and symptom development. Additionally, SRBSDV also exploits ethylene (ET) signalling (ET sig) to promote its proliferation and transmission. (**B**) Activation of phytohormones and PGRs signalling in response to virus attack. JA, salicylic acid (SA), nitric oxide (NO), and abscisic acid (ABA) participate in the activation of RNAi. SA and NO stimulate the expression of RNA‐dependent RNA polymerase (*RDR*) 1 to activate RNAi pathway, whereas ABA activates RNAi by inducing the expression of AGO proteins which in turn influences the expression of RDRs. Brassinosteroids (BRs) work together with SA in the induction of RBOH‐mediated H_2_O_2_ generation that subsequently shapes hypersensitive response (HR) and systemic acquired resistance (SAR). The induction of SA also results in the expression of pathogenesis‐related genes by expression of NPR1 and WRKY genes. SA, together with melatonin (Mel), also promotes the synthesis of phenolics and flavonoids in response to virus infection. Mel can affect endogenous NO and ET levels and regulate the expression of PR genes, which is also regulated by other PGRs such as Hydrogen sulfide (H_2_S), polyamines (PAs), and carrageenans, among others. Abbreviations: RSV, Rice stripe virus; TMV, tobacco mosaic virus; RDV, Rice dwarf virus; TCV, turnip crinkle virus; PCV, peanut clump virus, PTV, potyvirus; BYV, beet yellow virus; CaMV cauliflower mosaic virus, CuMV, cucumber mosaic virus; JAMYB, JA‐receptive MYB; DCL, dicer/dicer‐like protein; DRB, double‐stranded RNA binding proteins, AGO, Argonaute; RDR, RNA‐dependent RNA polymerase; NPR1, nonexpressor of pathogenesis‐related genes 1; PR, pathogenesis‐related. Figure created with BioRender.

Plant viruses tend to attenuate the RNAi mechanism to successfully infect the host tissues by producing silencing suppressors as a counter‐defense mechanism that interfere with the RNAi (Figure 3A) (Ding & Voinnet, 2007; Roth et al., 2004). Viral suppressors of RNA silencing (VSRs), which nearly all plant viruses have, provide compelling evidence for the antiviral character of RNA silencing. VSRs attenuate RNAi majorly by interfering with RISC's ability to cleave viral transcripts under the direction of vsiRNAs (Figure 3A). Especially certain VSRs attenuate the functions of AGO proteins, which are essential for the antiviral RNA silencing process (Carbonell & Carrington, 2015). For instance, the Cucumber Mosaic Virus 2b protein physically interacts with the PAZ domain of the AGO1 protein to inhibit RISC function (Duan et al., 2012). The majority of identified VSRs also perform various additional functions along with suppressing RNA silencing, which includes their functioning as coat proteins, proteases, motility proteins, helper components for viral transmission, or transcriptional regulators. Certain VSRs such as turnip crinkle virus (TCV) coat protein (CP or P38), peanut clump virus P15 protein, potyviral HcPro, and Beet Yellow Virus P21 protein, bind to short or long viral dsRNAs to sequester tiny RNA duplexes which prevents AGOs from assembling properly into RISC (Figure 3A) (Lakatos et al., 2006; Carbonell & Carrington, 2015). Moreover, a VSR from Cauliflower Mosaic Virus (CaMV) was shown to interact with the dsRNA binding protein 4 (DRB4), a crucial part of the DCL4 cleavage complex, to suppress DCL4 activity. This VSR, named translational trans‐activator protein P6, contains two nuclear localization signals and is essential for the virus infection. This virus‐originated P6 protein targets the DCL4 protein to inhibit the RNAi in plants, which facilitates its infection by evading the host's immune response (Figure 3A) (Laird et al., 2013).

In addition to these proteins associated with RNAi and other defence proteins such as PR‐proteins, some other proteins are also known to play key roles in plant resistance against virus infections. These proteins, also known as antiviral proteins, include ribosome inactivating proteins such as pokeweed antiviral protein that interfere with the viral protein synthesis by inhibiting the ribosomes (Di & Tumer, 2015). In addition, various secondary metabolites, including alkaloids such as 7‐deoxy‐trans‐dihydronarciclasine and bruceine‐D, and various quassinoids have been identified that exert direct antiviral activities (Zaynab et al., 2018). Additionally, the accumulation of various phytoalexins has also been observed in different plants in response to virus attacks, however, their exact roles in virus resistance are not very clear.

## 2 ROLE OF PHYTOHORMONES IN PLANT IMMUNITY AGAINST VIRAL INFECTIONS

### 2.1 Salicylic acid

SA, a phenolic compound and a key signalling molecule, plays a critical role in plant defence against viruses (Roychowdhury et al., 2024). SA is synthesized in plants either by the isochorismate pathway, which occurs in the chloroplasts or by phenylalanine ammonia‐lyase (PAL)‐mediated pathway, which takes place in the cytosol, both utilizing chorismite, the end product of the shikimate pathway. Pathogen invasion is known to trigger the SA biosynthesis, which subsequently induces key defence pathways involving NADPH oxidases or respiratory burst oxidase homologs (RBOHs)‐mediated ROS‐burst (Liu & He, 2016), expression of PR‐proteins, deposition of callose over the cell wall, and induction of the HR and SAR (Torres et al., 2006; wang et al., 2013). In Arabidopsis, pathogen‐induced SA accumulation is largely mediated by the isochorismate pathway (Nawrath & Métraux, 1999). SA is metabolized to catechol by the product of a bacterial *NahG* gene, which encodes for an SA hydroxylase, leading to a dramatic decrease in the plant SA content. SA‐deficient *nahG* transgenic lines exhibit enhanced susceptibility to pathogens, which confirms the key role of SA in plant resistance (Corina Vlot et al., 2009). SA‐deficient transgenic *NahG* potato plants showed reduced resistance and impaired SAR in response to Potato virus X (PVX) infection, which clearly suggests the critical role of SA in local and systemic defence against plant viral infections (Sánchez et al., 2010). It is critically important for plants to regulate SA biosynthesis to carefully balance the trade‐off between growth and defence. The long non‐coding RNA salicylic acid biogenesis controller 1 (*SABC1*) regulates plant growth and immunity by suppressing NAC3, which controls SA biosynthesis. *SABC1* recruits a repressive complex to reduce *NAC3* and *isochorismate synthase 1 (ICS1)* transcription, lowering SA levels and immunity in healthy plants. However, bacterial and viral infections result in a downregulation of *SABC1*, which activates defence responses, thereby balancing growth and immunity (C. Liu et al., 2022).

The role of SA in providing resistance against viruses was first highlighted almost five decades ago when Prof. R.F. White observed that injecting aspirin into the leaves of *Nicotiana tabacum* cv. *Xanthi‐nc* results in enhanced resistance to TMV. SA application triggered the expression of novel PR proteins, termed b_1_, b_2_, and b_3_, and was effective in reducing the number of local lesions by 90% (White, 1979). Subsequent experiments further validated this observation in various other plants, leading to decoding the molecular mechanism behind SA‐induced disease resistance. For instance, maize plants infected with sugarcane mosaic virus (SCMV) show enhanced SA production via phenylpropanoid pathway, which limits virus spread and disease severity. Accordingly, silencing of *ZmPAL* reduces SA accumulation, which decreases lignin production and enhances susceptibility towards the virus (Yuan et al., 2019). Another study showed that Coilin, a Cajal body protein, helps defend plants against tobacco rattle virus (TRV) by interacting with TRV's 16 K protein and relocating it to the nucleolus. This activates SA‐dependent defence pathways, limiting viral spread. Coilin deficiency or disrupted SA signalling enhances TRV infection, highlighting their crucial roles in antiviral defence (Shaw et al., 2019). In response to viruses, a number of studies have identified host factors that trigger SA‐signalling defensive mechanisms. For instance, the overexpression of glutathione biosynthesis genes and asparagine synthetase in tobacco provide resistance to TMV by activating SA‐signalling pathways (Zhu et al., 2021; N. Liu et al., 2022). The intriguing function of methyl salicylate (MeSA) in providing airborne protection against aphids and the viruses they carry was also discovered by another group of scientists using CMV infected *Nicotiana benthamiana*. CMV induces an SA burst that activates NAC2, triggering MeSA production via the salicylic acid carboxylmethyltransferase‐1 (*SAMT1*) gene. Infected plants emit this volatile compound, which nearby healthy plants perceive through salicylic acid‐binding protein‐2 (SABP2). SABP2 converts MeSA to SA, initiating a protective signalling cascade that deters aphids. To bypass this defence, the CMV1a protein relocates NAC2 to the cytoplasm for degradation, suppressing MeSA production and aiding viral transmission by enabling aphid attacks (Gong et al., 2023). Potato virus Y (PVY), another aphid‐transmitted virus, also produces a protein with a domain similar to that of CMV1a. This allows PVY to disrupt airborne defence mechanisms in a similar way by interacting with NAC2 (Gong et al., 2023). SA also helps plants defend against viruses by increasing lipid order and closing plasmodesmata. A study by (Huang et al., 2019) reveals that SA activates Remorin proteins to organize membrane nanodomains, reducing PD plasticity, limiting PD opening, and preventing virus spread. WRKY transcription factor NbWRKY40 enhances resistance to tomato mosaic virus (ToMV) in *Nicotiana benthamiana* by increasing SA levels. Overexpressing *NbWRKY40* reduces viral spread by promoting callose deposition at plasmodesmata, while its silencing makes plants more susceptible to ToMV (Jiang et al., 2021). The hypersensitive‐induced reaction (*HIR3*) gene family in *N. benthamiana* and rice is upregulated during rice stripe virus (RSV) infection. Silencing *HIR3* increases RSV accumulation, while its overexpression confers resistance to RSV and other pathogens. *HIR3* activates basal resistance through an *EDS1*‐ and SA‐dependent pathway, promoting cell death and pathogen defence (Li et al., 2019). The sulfotransferase (OsSOT1) encoded by the resistant rice *STV11* allele (*STV11‐R*) catalyzes the transformation of SA into sulphonated SA, a chemical that works more effectively in rice than SA, and prevents RSV replication and induces resistance to RSV (Wang et al., 2014).

SA can potentially impact all three primary phases of the virus infection cycle in infected plants, including their replication, intercellular transport, and long‐range movement (Table 1) (Alazem & Lin, 2015; Collum & Culver, 2016; Palukaitis et al., 2017). However, these SA effects are not generalized and seem to be virus and plant‐specific. For instance, Chivasa and co‐workers showed that SA‐induced resistance in tobacco plants to TMV was correlated with reduced virus RNA accumulation in virus‐inoculated plants (Chivasa et al., 1997). Experiments using GFP‐tagged TMV demonstrated that SA treatment restricts TMV movement across tobacco epidermal cells however, it exhibits only negligible effect on TMV replication. Notably, SA treatment also does not exhibit any direct effect in hindering the passage of viruses through plasmodesmata, as an increase in the size exclusion limit of plasmodesmata is observed due to a decrease in callose amount. This indirectly indicates that the amount of movement protein, the protein required for the local and systemic spread of the virus inside the plants, is insufficient to enable virus movement to epidermal cells, suggesting that SA inhibits virus movement from epidermal cells to mesodermal cells either by reducing biosynthesis or functions of movement protein (Murphy & Carr, 2002). In contrast, various other proteins have shown that SA application on the infected plants limits the virus infections such as potato virus X (PVX) (Falcioni et al., 2014) and turnip mosaic virus (TuMV) (Peng et al., 2013) by inhibiting viral replication. SA possibly interacts and inhibits the function of any target protein required for virus infection. Tian and co‐workers found that SA inhibits the replication of tomato bushy stunt virus (TBSV) via competitive interaction with cytosolic glycerol 3‐phosphate dehydrogenase (GAPDH). It was revealed that exogenously supplied SA interferes with the binding of cytosolic GAPDH to the negative (−) RNA strand of TBSV to suppress the virus replication (Tian et al., 2015). Plants employ RNAi as a primary strategy to combat viral infections, and accumulating data highlights a possible connection between RNAi and SA‐mediated defence (Carr et al., 2010). It has been reported that SA‐mediated repression of viral infection is stimulated by pre‐inducing genes related to the RNAi pathway (Zhao & Li, 2021). Viral infection and the presence of SA have been shown to trigger the expression of *RDR1*, an integral component of the RNAi pathway, in both *A. thaliana* and *N. tabacum* (Figure 3B) (Yu et al., 2003; Jovel et al., 2011; Hunter et al., 2013; Liao et al., 2013). In tomato, SA treatment markedly increased the expression of the Arabidopsis orthologs *ToDCL1*, *ToDCL2*, *ToRDR1*, and *ToRDR2*, which delayed the accumulation of ToMV RNA in infected plants (Campos et al., 2014). However, in the case of Arabidopsis, genes encoding for DCLs seem to be independent of SA‐induced resistance as SA treatment reduces CMV and TMV titres in *dcl2*, *dcl3*, and *dcl4* mutants (Yu et al., 2003). SA had a marginal but positive effect on the expression of *AGO2*, *AGO3*, and *AGO10* in *A. thaliana* (Alazem et al., 2019). It was also discovered that plants expressing *NahG*, when infected with plum pox virus (PPV), produced lower levels of siRNAs due to decreased accumulation of SA. Moreover, SA‐mediated defences against PPV were diminished by overexpression of the potyvirus silencing suppressor protein HC‐Pro (Alamillo et al., 2006). Autophagy has been identified as an additional layer in the interaction between SA and RNA silencing. A study on *A. thaliana* infected with CMV showed that elevated SA levels trigger autophagy, leading to the degradation of the 2b virulence factor and restricting viral RNA accumulation through RNA silencing (Shukla et al., 2022). Taken together, this upfront evidence confirms that the treatment of SA can orchestrate several antiviral defence strategies, including RNA silencing, autophagy, and initiation of the HR, which potentially limit the spread of the viruses by inhibiting their replication and limiting their cell‐to‐cell movement to reduce the virus load on the infected plants (Table 1) (Lee et al., 2016).

**TABLE 1:** Functional studies highlighting the role of phytohormones and PGRs signaling components in plant defense against viral infections.

S. No.	Phytohormone/PGR	Gene	Transgenic type	Plant	Virus	Outcome	Reference
1	Salicylic acid	*NahG*	Overexpression	*Solanum tubersosum*	Potato virus X (PVX)	Enhanced susceptibility	(Sánchez et al., 2010)
2	Salicylic acid	*NahG*	Overexpression	*Nicotiana tabacum*	Plum pox virus (PPV)	Enhanced susceptibility	(Alamillo et al., 2006)
3	Salicylic acid	(*Helper Component‐Proteinase*) *HC‐Pro*	Gain‐of‐function/ Overexpression	*Nicotiana tabacum*	Plum pox virus (PPV)	Enhanced susceptibility	(Alamillo et al., 2006)
4	Salicylic acid	STV11‐R	Knock‐in	*Oryza sativa*	Rice stripe virus (RSV)	Enhanced resistance	(Q. Wang et al., 2014)
5	Salicylic acid	*ZmPAL*	Knock down	*Zea mays*	Sugarcane mosaic virus (SCMV)	Enhanced susceptibility	(Yuan et al., 2019)
6	Salicylic acid	*WRKY40*	Knock down	*Nicotiana benthamiana*	Tomato mosaic virus (ToMV)	Enhanced susceptibility	(Jiang et al., 2021)
7	Salicylic acid	*HIR3*	Knock down	*Nicotiana benthamiana, Oryza sativa*	Rice stripe virus (RSV)	Enhanced susceptibility	(Li et al., 2019)
8	Jasmonic acid	*CORONATINE ‐INSENSITIVE 1* (*COI1*), *Allene oxide synthase* (*AOS*)	Knock down (Gene silencing)	*Nicotiana tabacum*	Tobacco mosaic virus (TMV)	Enhanced resistance	(Oka et al., 2013)
9	Jasmonic acid	*OsMYC2*	Knock‐out	*Oryza sativa*	Rice stripe virus (RSV)	Enhanced susceptibility	(Hu et al., 2020)
10	Jasmonic acid	*OsGSK2 (Glycogen synthase kinase)*	Knock down	*Oryza sativa*	Rice black‐streaked dwarf virus (RBSDV)	Enhanced susceptibility	(He et al., 2020)
11	Jasmonic acid	(*TEOSINTE BRANCHED*/*CYCLOIDEA*/*PCF*) *TCP21*	Knock‐down	*Oryza sativa*	Rice ragged stunt virus (RRSV)	Enhanced susceptibility	(Zhang et al., 2016)
12	Abscisic acid	*WRKY8*	Knockout	*Arabidopsis thaliana*	Crucifer infecting‐tobacco mosaic virus (TMV‐cg)	Enhanced susceptibility	(Chen et al., 2013)
13	Abscisic acid	*ABA2*	Knockout	*Arabidopsis thaliana, Nicotiana benthamiana*	Bamboo mosaic virus (BaMV), Cucumber mosaic virus (CMV)	Enhanced resistance	(Alazem et al., 2013)
14	Abscisic acid	*aao3*, *abi1‐1*, *abi3‐1*, and *abi4‐1*	Knockout	*Arabidopsis thaliana, Nicotiana benthamiana*	Bamboo mosaic virus (BaMV), Cucumber mosaic virus (CMV)	Enhanced susceptibility	(Alazem et al., 2013)
15	Abscisic acid	*OsABIL2*	Overexpression	*Oryza sativa*	RBSDV	Enhanced resistance	(Xie et al., 2018)
16	Ethylene	(1‐aminocyclopropane‐1‐carboxylate synthase) *acs1*, (ethylene‐response transcription factor) *erf106*, (ethylene insensitive) *ein2*	Knockout	*Arabidopsis thaliana*	Crucifer infecting‐tobacco mosaic virus (TMV‐cg)	Enhanced resistance	(Chen et al., 2013)
17	Ethylene	*ClACO5* (*ACO‐*ACC Oxidase)	Knock down	*Citrullus lanatus*	Cucumber green mottle mosaic virus (CGMMV)	Enhanced susceptibility	(Liu et al., 2023)
18	Ethylene	AGD2‐LIKE DEFENCE RESPONSE PROTEIN 1 (*ALD1*)	Knock down	*Nicotiana benthamiana*	Turnip mosaic virus (TuMV)	Enhanced susceptibility	(S. Wang et al., 2019)
19	Brassinosteroid	*BRI1‐associated receptor kinase 1 (BAK1); bak1‐4, bak1‐5*	Knockout	*Arabidopsis thaliana*	TCV, oilseed rape mosaic virus, and TMV	Enhanced susceptibility	(Julie KØrner et al., 2013)
20	Brassinosteroid	*NbBRI1*, *NbBSK1*, *NbDWARF, NbBAK1* and *NbBIK1*	Knock down	*Nicotiana benthamiana*	TMV	Enhanced susceptibility	(Deng et al., 2016)
21	Brassinosteroid	*NbBES1/BZR1*	Knock down	*Nicotiana benthamiana*	TMV	Enhanced resistance	(Deng et al., 2016)

### 2.2 Jasmonate

JA is another phytohormone that plays a pivotal role in plant disease resistance. It is an oxygenated fatty acid (oxylipin), the biosynthesis and modification of which takes place in three cellular compartments, including chloroplast, peroxisome, and cytoplasm (Gupta et al., 2016). In the chloroplast, unsaturated fatty acids are converted to 12‐oxo‐phytodienoic acid (12‐OPDA) or deoxymethylated vegetable dienic acid (dn‐OPDA), which is then transformed into JA in the peroxisome. Chemical processes in the cytoplasm convert JA into a variety of compounds, including methyl jasmonate (MeJA), jasmonate isoleucine (JA‐Ile), cis‐jasmone (CJ), and 12‐hydroxyjasmonic acid (12‐OH‐JA) (Ruan et al., 2019). In the case of virus infections, the role of JA biosynthesis and signalling seems to be complex and is not yet fully understood. Although many studies have shown alterations in JA concentrations and expression of JA signalling genes in plants during virus attacks, the exact role of JA remained largely enigmatic (Alazem & Lin, 2015; Collum & Culver, 2016; Yang et al., 2020). In the E3 ubiquitin‐ligase SKP1‐Cullin‐F‐box complex, the F‐box protein CORONATINE INSENSITIVE1 (COI1), which acts as a receptor of jasmonic acid‐isoleucine (JA‐Ile), and JASMONATE ZIM (JAZ) repressor proteins are primarily responsible for the activation of JA signalling (Sheard et al., 2010). JA‐signalling is negatively regulated by JAZs. To prevent the activation of the JA pathway in resting cells, JAZ repressor proteins, the adaptor protein NOVEL INTERACTOR OF JAZ (NINJA), and the recruited corepressor TOPLESS (TPL) bind with positive transcriptional factors, including basic helix–loop–helix MYCs (Pauwels et al., 2010). The JA‐Ile signal binds to COI1 in JA‐stimulated cells, activating downstream gene expression and immunity, which is followed by the proteasome's degradation of JAZ (Lorenzo et al., 2004; Pauwels & Goossens, 2011; Kazan & Manners, 2013). A multi‐layered defence against external stress is integrated by these transcriptional regulators. A typical marker for characterizing the jasmonate‐dependent defence responses is the Arabidopsis *PDF1.2* gene, which codes for a plant defensin (Brown et al., 2003).

**FIGURE 4:**
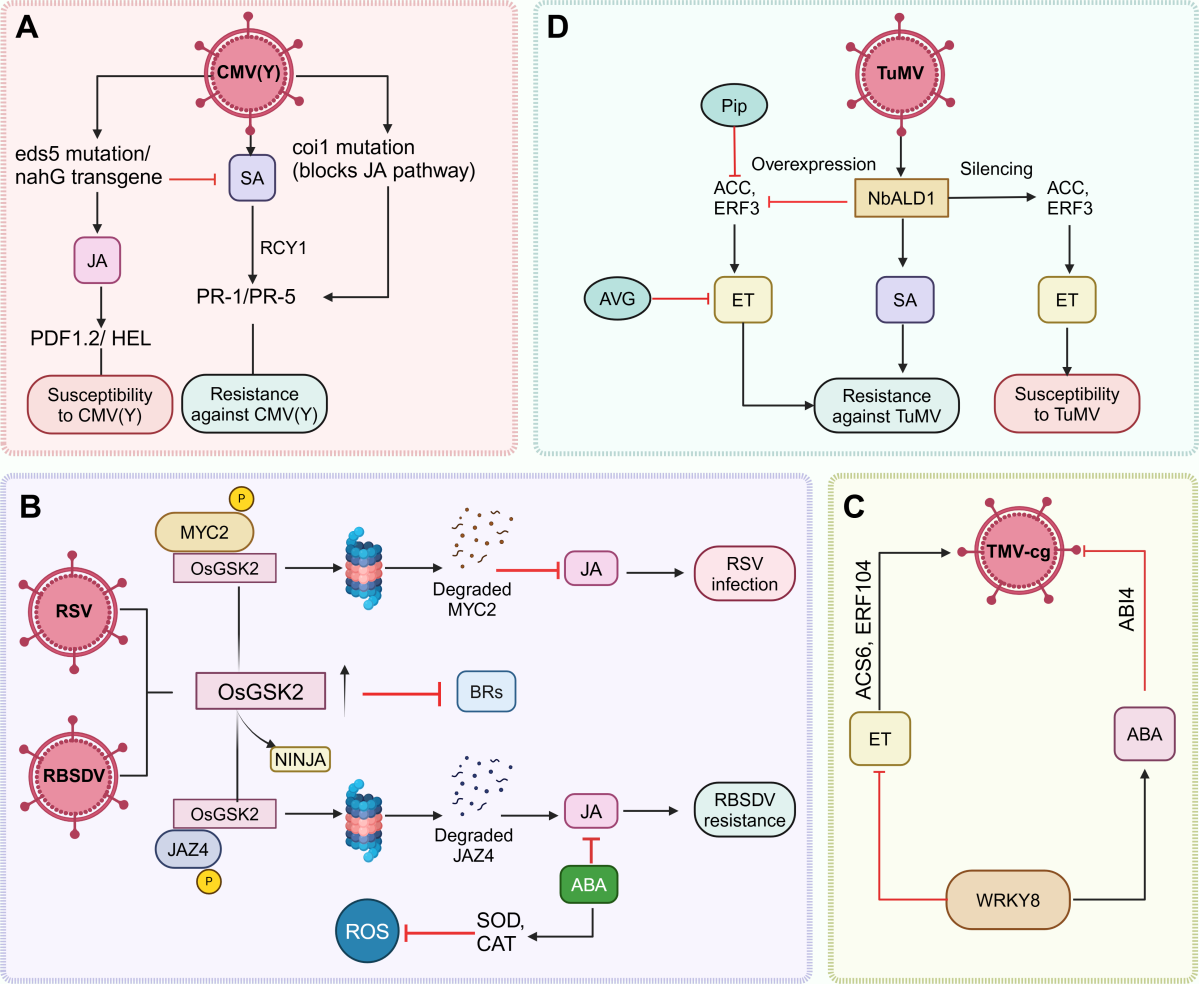
**Crosstalk among different phytohormones in shaping plant defence response against viruses. (A) A diagram showing antagonism between SA and JA in response to a yellow strain of cucumber mosaic virus CMV(Y) infection in Arabidopsis conferred by the dominant RCY1 allele**. Resistance and the expression of *PR‐1* and *PR‐5* are partially compromised by the *eds5* mutation and the *nahG* transgene, which block salicylic acid (SA) accumulation. The *coi1* mutation, which disrupts jasmonic acid (JA) signalling, restores *PR‐1* and *PR‐5* expression and enhances resistance in *eds5 coi1 RCY1* plants. Elevated expression of *PDF1.2* and *HEL* in *eds5 RCY1* plants indicates antagonistic interactions between SA and JA in modulating *RCY1*‐mediated resistance. **(B) Jasmonic acid (JA)‐Brassinosteroid (BR) crosstalk and the role of OsGSK2 in modulating BR and JA signalling in rice upon infection by Rice stripe virus (RSV) and Rice black‐streaked dwarf virus (RBSDV)**. RSV infection upregulates *OsGSK2*, leading to the degradation of *MYC2*, inhibiting JA signalling and promoting RSV infection. In contrast, *OsGSK2* accumulation during RBSDV infection degrade *JAZ4*, which activates JA signalling, disrupting its interaction with NINJA and enhancing resistance. *OsGSK2* negatively regulates BR signalling. ABA is shown to negatively impact defence against RBSDV by suppressing JA‐mediated resistance and reducing reactive oxygen species (ROS) accumulation by inducing antioxidant enzymes. **(C) Role of WRKY8 in balancing ABA‐ET interaction against TMV‐cg in Nicotiana benthamiana**. TMV‐cg infection inhibits WRKY8 expression. WRKY8 regulates the defence response to TMV‐cg by activating ABI4, promoting ABA signalling, and repressing ET‐related genes like ACS6 and ERF104, reducing ET accumulation. **(D) Crosstalk between Salicylic acid (SA) and Ethylene (ET) pathways and regulatory role of NbALD1 in the resistance of *Nicotiana benthamiana* to Turnip mosaic virus (TuMV)**. NbALD1 enhances resistance by activating the SA pathway while suppressing the ET pathway. Silencing NbALD1 increases the levels of ET precursors ACC and ERF3, leading to higher ET production and increased susceptibility to TuMV. Conversely, overexpressing *NbALD1* or exogenously applying pipecolic acid (Pip) suppresses ET accumulation, enhances the SA‐mediated defence response, and improves resistance against TuMV. AVG, an ET biosynthesis inhibitor, also reduces ET accumulation, supporting the role of ET in viral susceptibility. Figure created with BioRender.

Antagonism between JA and SA appears to frequently play a role in regulating viral defence response while JA, together with ET, typically control induced systemic resistance (ISR), another significant type of systemic defence response in plants (Figure 4A) (Yang et al., 2015, Meng et al., 2019). The antagonism between SA and JA signalling is shown to activate a leucine‐rich repeat‐type resistance gene, *RCY1*, which induces resistance to CuMV strain Y/CMV(Y) in Arabidopsis (Figure 4A) (Takahashi et al., 2004). Moreover, N‐gene‐mediated resistance in tobacco plants to TMV is shown to be because of the attenuation of JA biosynthesis and signalling. Accordingly, suppressing the JA biosynthesis or signalling either by silencing the JA receptor *COI1* or JA biosynthetic enzyme, *allene oxide synthase* (*AOS*), enhances SA accumulation, which significantly decreases the TMV accumulation in N‐gene resistant tobacco (Oka et al., 2013). Additionally, exogenous application of MeJA to these N‐gene‐resistant tobacco plants promotes systemic viral movement inside the host, which confirms a negative role of JA in plant resistance against viral infections.

Contrary to these reports, other studies have shown that JA signalling is activated during viral attacks and is positively associated with resistance. For instance, endogenous JA levels have also been shown to increase substantially in tobacco and potato plants during incompatible plant‐virus interactions, which indicates a potential role of JA in imparting resistance (Dhondt et al., 2000; Kovac et al., 2009). Exogenous MeJA treatment enhances resistance to Mungbean yellow mosaic India virus (MYMIV) in *Vigna mungo* by stabilizing membranes, maintaining ROS balance, and upregulating defence genes (Chakraborty & Basak, 2019). JA pathway is also activated by RSV infection, with its coat protein (CP) inducing JA‐related defences against the virus. However, JA also attracts the small brown planthopper (SBPH), enhancing RSV transmission. This highlights the dual role of JA in defending against RSV and facilitating its spread via the vector (Han et al., 2020). In plants infected with RBSDV, OsGSK2 enhances antiviral defence by phosphorylating and destabilizing OsJAZ4, a negative regulator of JA signalling, disrupting its interactions with OsNINJA and OsJAZ11. This activates JA signalling, promoting defence. Conversely, the BR pathway stabilizes OsJAZ4 via OsCOI1, suppressing JA signalling. OsGSK2 thus links BR and JA pathways for defence regulation (He et al., 2020). Whereas in another experiment with RSV‐infected plants, BRs and JA synergistically enhance rice resistance to RSV, with BR‐mediated resistance depending on an active JA pathway. RSV suppresses BR signalling by increasing OsGSK2, which degrades OsMYC2, a JA regulator, weakening defences (Figure 4B). This reveals a novel BR‐JA crosstalk mechanism for antiviral resistance (Hu et al., 2020).

Exogenous JA treatment is also effective in reducing viral load on multiple plants, including *Arabidopsis* where the DNA titer of a geminivirus, beet curly top virus (BCTV), is decreased as a result of continuous JA treatment. These findings indicate that JA plays a positive role in plant resistance against geminiviruses, and suppression of the JA response may be essential for virus infection (Lozano‐Durán et al., 2011). These results were further supported by the identification of a geminivirus C2 protein during compatible host‐geminivirus interactions. This geminivirus C2 protein interacts with the catalytic subunit of COP9 signalosome (CSN) of host plants to hijack and reduce SCF ubiquitin ligase activity that suppresses JA signalling (Figure 3B). These results confirm that this virus has developed a method for modifying host resistance by C2 targeting of SCF ubiquitination (Lozano‐Durán et al., 2011). Another virus, rice ragged stunt virus (RRSV), has developed a completely different strategy to attenuate the JA signalling. RRSV infection leads to the suppression of *TEOSINTE BRANCHED/CYCLOIDEA/PCF* (*TCP*) expression and accumulation of miR319 in rice. Notably, overexpression of miR319 or suppression of *TCP21* results in reduced expression of JA biosynthetic genes and, accordingly, reduced JA levels, leading to an increased sensitivity to RRSV and the development of disease‐like traits. These results show that miR319 and *TCP21* are associated with JA biosynthesis and signalling in rice. A study showed that histone deacetylase, OsHDA706 deacetylates and stabilizes OsLOX14, a key enzyme in JA biosynthesis, enhancing JA accumulation and antiviral defence in rice. The RSV protein P2 disrupts this OsHDA706‐OsLOX14 interaction, reducing JA levels and promoting viral infection (Yang et al., 2024). Additionally, rice plants treated with MeJA display reduced RRSV accumulation, further confirming the positive role of JA in plant defence against geminivirus infections (Zhang et al., 2016). Together with these studies, emerging evidence also suggests a positive role of JA in inducing RNAi during virus infections. For instance, a recent study revealed that JA signalling transcriptionally activates an argonaute, *AGO18*, through JAMYB, leading to the upregulation of RNA silencing components and promoting antiviral defence in rice against RSV (Yang et al., 2020). However, additional experiments are required to confirm the exact role of JA in RNAi further. Since RNAi is also known to be activated in response to SA and both SA and JA antagonize each other, it is highly unlikely that both of these phytohormones participate synergistically in the activation of RNAi, which is a key antiviral response in plants. Taken together, these results suggest that JA controls plant defence through complex interactions with many signalling networks that are influenced by other phytohormones, such as SA, rather than by linear routes (Ballaré, 2011; Pieterse et al., 2009). The plant seems to activate the best defensive response to a particular invader through the combination of antagonistic or synergistic interactions between JA and SA signalling networks, which greatly increases plant defence in response to invading viral pathogens.

### 2.3 Abscisic acid

ABA is a sesquiterpenoid that was first identified in abscising cotton fruit as a growth inhibitor (Abscisin II) (Addicott, 1983). In plants, ABA is biosynthesized from isopentenyl diphosphate (IPP), which is produced by the methylerythritol phosphate (MEP) pathway, principally in plastids from glyceraldehyde 3‐phosphate and pyruvate. This causes the intermediates phytoene and lycopene to be produced in succession. Zeaxanthin is the first oxygenated carotenoid that is produced when lycopene is subjected to cyclization and hydroxylation (Nambara & Marion‐Poll, 2005). ABA is known to regulate various physiological processes such as the closing of stomata, floral induction, seed dormancy, inhibition of germination, and stress responses, including salinity, drought, and pathogen infection (Mauch‐Mani & Mauch, 2005; Wasilewska et al., 2008; Finkelstein, 2013). Although the role of ABA in viral resistance is not well established, accumulating data suggest its positive role in imparting resistance. At first, Rezzonico et al., 1998 showed that ABA can limit virus spread by inhibiting *β*‐1,3‐glucanase, which is responsible for callose degradation. Subsequent experiments suggested that ABA imparts resistance to viral pathogens principally by promoting callose deposition at plasmodesmata (PD) (Iglesias & Meins, 2000; De Storme & Geelen, 2014) and by triggering the RNAi (Alazem & Lin, 2015; Alazem et al., 2017). The R gene‐mediated resistance to the soybean mosaic virus (SMV) is exclusively associated with the upregulation of a subset of type 2C protein phosphatase (PP2C) genes (Figure 3B). The identification of SMV CI protein by the plants triggers the accumulation of ABA and the deposition of callose, mediated by PP2C, which inhibits the virus movement (Seo et al., 2014). In addition, ABA also participates in stomatal closure, one of the initial immune responses and an efficient strategy to inhibit the entry of pathogens inside the host tissues. Apart from ABA, the signalling pathway of stomatal closure involves protein kinases, secondary messengers, receptors, ion channels, ion efflux, and turgor loss in guard cells. OST1 is one of the main kinases that activates NADPH oxidase and increases the ROS levels in guard cells. An increase in OST1 kinase activates RBOH D/F, which increases ROS/NO/Ca^2+^ levels, whereas Ca^2+^ dependent CDPKs activate slow anion channel 1 (SLAC1), S‐type anion channel 3 (SLAH3) and K^+^ channels which to promote ion efflux from guard cells, thereby inducing the stomatal closure (Bharath et al., 2021). Subsequent evidence on the positive role of ABA in imparting virus resistance was derived from the observations that the ABA‐deficient Arabidopsis mutants exhibit higher systemic accumulation of crucifer‐infecting tobacco mosaic virus (TMV‐cg) (Chen et al., 2013) and enhanced susceptibility to bamboo mosaic virus (BaMV) (Alazem et al., 2013). It was revealed that *WRKY8* regulates *Arabidopsis* defence against TMV‐cg by activating ABA signalling while suppressing ET signalling (Figure 4C). Mutations in *WRKY8* or ABA genes promote TMV‐cg spread, highlighting *WRKY8*'s role in balancing ABA and ET pathways. The study by (Alazem et al., 2013) shows that *ABA2* supports Bamboo mosaic virus (BaMV) and CMV accumulation in plants, while downstream ABA pathway genes contribute to resistance. Another experiment reveals that ABA negatively impacts rice defence against RBSDV. ABA suppresses the JA‐mediated resistance and reduces ROS accumulation by inducing antioxidant enzymes like SOD and CAT. (Xie et al., 2018). The study used ABA‐insensitive mutants, specifically *ABIL2‐ox‐4* and *ABIL2‐ox‐6*, which overexpress the *OsABI‐LIKE2* (*OsABIL2*) gene. *OsABIL2* encodes a type 2C protein phosphatase that negatively regulates ABA signalling. The results showed that blocking ABA signalling enhances rice resistance to RBSDV, confirming that ABA signalling negatively modulates immunity to the virus (Xie et al., 2018). Taken together, these results suggest that the effects of ABA vary depending on the pathosystem and the infection stage (Ton et al., 2009). ABA signalling, specifically if activated during the early stages of viral infection, can enhance resistance by closing stomata and by inducing callose deposition over the cell walls (Ton et al., 2009; Ellinger & Voigt, 2014). However, if ABA signalling is activated later in the infection process once the pathogen successfully colonizes the plant tissues, it can impair plant defence by competing with other hormone pathways, such as ET, JA or SA (Anderson et al., 2004; Yasuda et al., 2008). Therefore, early induction of ABA signalling is crucial for ABA‐mediated activation of plant defence and for restricting pathogen invasion.

Although there seems to be an antagonistic relationship between ABA and SA, they both participate in the activation of RNAi (Alazem et al., 2019). The RNAi pathway plays a major role in the ABA‐dependent resistance against BaMV. ABA is known to induce AGO2 and AGO3, but not AGO1, which is thought to be responsible for inducing RNAi against BaMV (Figure 3B) (Alazem et al., 2017; Alazem et al., 2013). Moreover, ABA is known to function upstream of SA as ABA needs activation of AGO2, which requires SA. ABA and AGO2 have also been shown to provide resistance against potato virus X (PVX) in *A. thaliana*, which suppresses PVX replication (Jaubert et al., 2011). Additionally, the ABA biosynthetic mutants *aao3* and *aba2‐1* exhibit downregulation of multiple *AGO* genes, which clearly suggests that ABA is required for the virus‐induced induction RNAi by triggering the expression of *AGO* genes (Alazem et al., 2013, 2019; Song et al., 2016). Notably, mutants in many RNAi genes also exhibit hypersensitivity to ABA, as reported for the *A. thaliana* mutant lines of *HYPONASTIC LEAVES (HYL1*), *HUA ENHANCER (HEN1*), *AGO1*, or *DCL* (ZHANG et al., 2008), further confirming a possible relationship between ABA and RNAi components and that these RNAi associated genes exert a regulatory feedback loop on ABA (Alazem & Lin, 2020).

### 2.4 Ethylene

ET, the first gaseous phytohormone to be identified in plants, plays a critical role in senescence and defence responses predominantly against necrotrophic pathogens (Pieterse & Dicke, 2007; Koornneef & Pieterse, 2008). Sulfur‐containing amino acid methionine functions as a precursor for ET biosynthesis, which is converted to S‐adenosylmethionine (SAM) by S‐adenosylmethionine synthetase (Riyazuddin et al., 2020). SAM is then converted into 5‐deoxy‐5‐methylthioadenosine (MTA) and 1‐aminocyclopropane‐1‐carboxylic acid (ACC) by enzyme1‐aminocyclopropane‐1‐carboxylase (ACS). ACC oxidase finally converts ACC into ET, CO_2_, and cyanide (ACO) (Adams & Yang, 1979). When a virus infects a plant, ET biosynthesis is induced and is linked to the development of symptoms (Marco & Levy, 1979; Ohtsubo et al., 1999; Geri et al., 2004). However, the exact role of ET in plant defence against viruses is debatable. In certain interactions, it fosters disease progression, while in others, it imparts resistance (Casteel et al., 2015; S. Zhao et al., 2017).

Multiple studies have reported alterations in ET levels in plants during virus attacks. For instance, tobacco plants, when infected with chilli veinal mottle virus (ChiVMV), display elevated ET and JA levels along with enhanced expression of N‐gene at the early stages of infection (Zhu et al., 2014). However, in the case of tomato, enhanced systemic resistance against CMV in response to biocontrol agent *Trichoderma harzianum* strain T22 treatment is mediated by reduced ET levels. In comparison to healthy controls, T22 application either prior to or concurrently with CMV results in a significant increase in the foliar JA/ET ratio and SA levels, which provides resistance (Vitti et al., 2016). Another experiment reveals that *NbALD1* enhances resistance to turnip mosaic virus (TuMV) in *Nicotiana benthamiana* by boosting the SA pathway while suppressing the ET pathway. Silencing *NbALD1* increases ET precursor ACC and susceptibility, while overexpressing *NbALD1* or applying pipecolic acid (Pip) reduces ET levels, enhances SA‐mediated resistance, and confirms a negative role of ET, suggesting the possible crosstalk between the two hormones during virus infection (Figure 4D) (Wang et al., 2019). In the same way, ACC spraying on *Phaseolus vulgaris* prior to white clover mosaic potexvirus (WClMV) infection decreased viral titres. Furthermore, JA and SA spraying contributed to reducing virus levels (Clarke et al., 1998). These alterations in ET levels suggest its possible involvement in plant‐virus interactions, however, since these alterations are accompanied by similar changes in other phytohormones such as JA and SA, it can be speculated that ET exhibits an indirect role in plant‐virus interactions by interacting with other phytohormones and signalling molecules. However, a recent report revealed a direct role of ET in the plant‐virus interaction in the case of Southern rice black‐streaked dwarf virus (SRBSDV), which is predominantly transmitted by planthoppers. SRBSDV‐encoded P6 protein plays a crucial role in regulating ET signalling in rice by coordinating viral infection and transmission by insect vectors. In the early phase of SRBSDV infection, the P6 interacts with OsRTH2 in the cytoplasm, leading to the activation of ET signalling, which enhances the proliferation of SRBSDV within the host plant while repelling the insect vectors to reduce infestation, particularly planthoppers. However, P6 moves into the nucleus and interacts with OsEIL2, an essential ET signalling transcription factor, during the late phases of infection. By preventing OsEIL2 from dimerizing, the P6‐OsEIL2 association inhibits ET signalling and promotes viral transmission by drawing in the insect vector (Figure 3B) (Zhao et al., 2022). This study reveals how viruses hijack plant phytohormone signalling to manipulate their replication and suppress the host's immune response. Another study utilizing the P6 protein further evidenced the role of ET signalling in symptoms development during virus infections. Geri et al., 2004 reported that transgenic Arabidopsis plants containing P6 protein develop milder and delayed symptoms during CaMV infection as compared to the WT plants. The P6 transgenic line was nearly entirely ET insensitive, whereas infected WT plants were just slightly ET insensitive. Based on these results, it can be speculated that the interactions among P6 and specific components of the ET pathway are necessary for the development of the symptoms in host plants in response to CaMV infection. Likewise, accumulation of ET precursor, ACC, has been observed locally around necrotic patches in *N. tabacum* plants that are resistant to TMV or TNV, suggesting a potential role of ET in lesion formation (de Laat & van Loon, 1983; Ohtsubo et al., 1999). However, ACC, when sprayed prior to infection, prevented lesion formation (Ohtsubo et al., 1999; Knoester et al., 2001; de Laat & van Loon, 1983). *Citrullus lanatus* (Watermelon) responds to cucumber green mottle mosaic virus (CGMMV) infection by producing ET, regulated by the *ClWRKY70*‐*ClACO5* module. *ClWRKY70* activates *ClACO5*, a key ET biosynthesis gene, to enhance ET production and virus resistance. Accordingly, silencing of *ClACO5* increases susceptibility while its overexpression imparts resistance (Liu et al., 2023). In contrast, Chen et al., 2013 revealed that ET biosynthesis and signalling mutants exhibit enhanced resistance to TMVcg, and the application of ET precursor increased the accumulation of TMVcg, indicating a negative involvement of ET in plant resistance against viruses (Chen et al., 2013). These results suggest that the time of treatment has a significant impact on plant defence against viral infection, even if endogenous JA and ET have antagonistic effects on SA‐mediated defence against viruses. However, the overall data on the ET's‐mediated regulation of plant defence are scarce, and more studies are required to establish the functional roles of ET in plant‐virus interactions.

## 3 ROLE OF EMERGING PGRS IN PLANT IMMUNITY AGAINST VIRAL INFECTIONS

### 3.1 Brassinosteroids

Brassinosteroids (BRs), a group of polyhydroxy steroid hormones that were first isolated from pollens of *Brassica napus*, are known to positively regulate defence responses against viruses (Khripach et al., 2000; Xia et al., 2009). Brassinolide (BL), the most important BR, is known to induce disease resistance in tobacco plants against TMV. BL‐treated tobacco plants exhibit enhanced resistance to the viral pathogens in an SA‐independent manner. BL treatment does not affect the expression of SA‐induced genes such as acidic and basic PR‐proteins, implying that BL‐mediated defence is different from SAR (Nakashita et al., 2003). This BR‐induced resistance against TMV involves activation of MEK2‐SIPK (salicylic acid‐induced protein kinase), which further induces RBOHB (respiratory burst oxidase homolog protein B)‐dependent ROS burst, thereby imparting resistance. BES1/BZR1 serves as an important mediator of BR signalling and participates in growth‐immunity tradeoff by reducing RBOHB‐dependent ROS production. Further, silencing of BR biosynthesis or signalling genes, including *NbDWARF, NbBRI1, NbBSK1, NbBAK1*, or *NbBIK1*, results in enhanced susceptibility of tobacco plants to TMV infection (Deng et al., 2016). Likewise, Zhang et al., 2015, demonstrated that Arabidopsis plants treated with BR exhibit enhanced resistance to CMV. Additionally, BR treatment also alleviates photosystem damage, enhances the activity of antioxidant enzymes, and induces the expression of defence‐associated genes in Arabidopsis plants under virus attack. Moreover, BR treatment also improves photosynthetic efficiency and expression of various defence‐related genes such as *PR1*, *PR2*, *MAPK3*, *MAPK6*, and *WRKY30* that are known to be activated downstream of SA signalling (D.‐W. Zhang et al., 2015; Xia et al., 2009).

Similar to the JA, BR signalling is also emerging as a crucial component of plant defence response against geminiviruses. In Arabidopsis, the geminivirus C4 protein is shown to interact with BRASSINOSTEROID‐INSENSITIVE 2 (BIN2), a negative regulator of BR signalling; however, the relevance of this interaction could not be determined (Piroux et al., 2007). BRI1‐associated receptor kinase 1 (BAK1) functions as a co‐receptor of BR and is also known to play important functions in plant‐virus interactions, especially against RNA viruses. *bak1‐4* and *bak1‐5* Arabidopsis mutants show enhanced accumulation of TCV, oilseed rape mosaic virus (ORMV), and TMV as compared to the wild‐type plants, indicating that BAK1 functions as a positive regulator of plant defence responses against various viruses (Julie KØrner et al., 2013). BR is also known to antagonize JA signalling, therefore, BR treatment does not always have a beneficial impact on plants against virus resistance. For instance, it has recently been discovered that BR makes rice plants more vulnerable to the rice black‐streaked dwarf virus (RBSDV) infection. This enhanced susceptibility of rice plants to RBSDV is due to the BR‐mediated suppression of JA‐induced defence response. Additionally, rice plants infected with RBSDV exhibit reduced expression of BR biosynthesis (*OsCPDs* and *OsDWF4*) and signalling (*OsBRI1* and *OsBZR1*) genes and enhanced expression of JA biosynthetic genes (He et al., 2017). In addition to indicating an antagonistic relationship between BR and JA, these results also suggest that the virus possibly exploits the BR signalling of host plants to suppress JA signalling, which subsequently promotes infection.

### 3.2 Melatonin

Melatonin (N‐acetyl‐5‐methoxytryptamine) is a multifunctional molecule derived from tryptophan and produced by animals, plants, bacteria, and fungi (Gupta 2023). Melatonin was first discovered in the bovine pineal gland (Sharif et al., 2018; Arnao & Hernández‐Ruiz, 2015) in 1958 and has long been recognized for its hormonal role in animals. It has also been utilized to fight viral diseases in both animals and humans in recent years, for example, in the treatment of the Venezuelan equine encephalomyelitis (VEE) virus (Bonilla et al., 2004) and Ebola virus (Tan et al., 2014) in humans. Several studies have also investigated the use of melatonin as a potential treatment for COVID‐19, either as an active or adjuvant drug at different stages of treatment (Cardinali et al., 2020; Zhang et al., 2020). In recent years, the presence of melatonin has also been reported in plants and is turning out to be a multi‐regulatory molecule with pleiotropic effects (Dubbels et al., 1995; Hattori et al., 1995; Kolar et al., 1995; Arnao et al., 2022). Phytomelatonin plays a crucial role in plant growth and development (Arnao & Hernández‐Ruiz, 2015; Sharif et al., 2018) and in plant protection against diverse stresses, including biotic stresses (Gupta, 2023). Considering virus infection in plants, only a few such reports are available where exogenous melatonin treatment has been used to reduce the severity of viral disease symptoms as well as the accumulation of the virus in the plants. Melatonin treatment upregulates the expression of several genes associated with plant defence, such as those encoding for PR proteins and enzymes involved in the biosynthesis of SA and JA. Chen et al., 2019 showed that exogenous melatonin application on *in vitro* cultured apple shoots can help in the eradication of apple Stem Grooving Virus (ASGV) in a dose‐dependent manner. Application of 15 μm melatonin to the 4‐week‐old shoots led to the generation of 95% virus‐free plants following shoot tip culture (Chen et al., 2019). Melatonin also provides resistance to rice stripe virus (RSV) through a NO‐dependent pathway. Exogenous application of melatonin or NO to rice reduces the incidence of RSV infection, while the application of NO scavenger, cPTIO, reverses the positive effect of melatonin. Moreover, the application of melatonin could significantly increase the NO levels, however, a NO donor does not affect melatonin levels. These results indicate that NO serves as a mediator and a downstream signalling component in the process of melatonin‐triggered rice resistance to RSV. The application of melatonin, together with NO donor, is known to induce the expression of defence genes *OsPR1b* and *OsWRKY45* in rice (Lu et al., 2019). More recently, Zhao et al., 2019 demonstrated that melatonin treatment can limit local and systemic TMV infections in *Nicotiana glutinosa* and *Solanum lycopersicum*, respectively. Exogenous application of melatonin enhances the SA levels in the TMV‐infected *S. lycopersicum* seedlings, indicating melatonin functions by inducing SA‐mediated defence responses in plants. These results were further supported in other pathosystems such as eggplant and alfalfa Mosaic Virus (AMV) where cotreatment of melatonin and SA substantially reduces the virus concentration and virus‐induced oxidative (Sofy et al., 2021). Melatonin and SA treatments also induce the accumulation of flavonoids and phenolics that function as antioxidants, blocking the attachment and penetration of viruses into cells and inducing defence systems inside host cells (Friedman, 2007). These results demonstrate the efficacy of melatonin treatment in inducing plant defence against a number of viral pathogens. However, the evidence gathered so far suggests that melatonin majorly functions by inducing SA and/or NO signalling‐mediated defence responses (Figure 3B). As the number of studies involving melatonin in plant‐virus interactions is currently limited, more data is required to completely understand the melatonin‐induced defence mechanism in plants.

### 3.3 Nitric oxide

NO is a small, diatomic, gaseous molecule that functions as a signalling molecule for plants under both normal and stressful circumstances, with a variety of physiological, biochemical, and molecular implications. NO treatment has been shown to improve plant resistance against a number of viral pathogens. For instance, tobacco plants treated with NO‐donor show significantly reduced TMV lesions. Notably, only upper leaf lesions are reduced in response to NO‐donor treatment, while not much effects are observed in the lower tobacco leaves. It implies that the lower leaves might be producing the signal(s) needed for the development of SAR in the upper leaves (Song & Goodman, 2001). Moreover, plants infected with viruses also exhibit biosynthesis of H_2_O_2_ and NO, which activate SA‐mediated defence responses (Figure 3B) (Wendehenne et al., 2004; Torres et al., 2006; Asai & Yoshioka, 2009). This NO‐mediated activation of SAR is possibly mediated through mitochondrial alternative oxidase (AOX), as NO‐mediated AOX induction has been reported in tobacco in response to TMV, which then triggers systemic defence responses (Fu et al., 2010).

NO is also known to activate RNAi by triggering the RDR1, downstream of NO‐mediated H_2_O_2_ production (Figure 3B) (Liao et al., 2013). Another study suggests that NO controls the expression of calreticulin and *β*‐1,3‐glucanase to contribute to the resistance to TMV caused by chitosan oligosaccharide (Zhang et al., 2019). Similar results have also been reported in response to various other viruses, such as RBSDV where the application of NO donor enhances endogenous NO levels that greatly lowers the disease incidence (Lu et al., 2020). NO also seems to collaborate with BRs to limit the viral loads on the plants. Zou and colleagues reported that *Arabidopsis* plants treated with BRs display NO accumulation and significantly reduced accumulation of viruses in the plants. In contrast, plants pre‐treated with NO scavenger cPTIO or nitrate reductase (NR) inhibitor tungstate, exhibit higher viral replication and enhanced virus‐induced damage. This suggests that NO also plays a crucial role in the virus resistance induced by BR treatment (Zou et al., 2018).

### 3.4 Polyamines

Polyamines (PA) are organic, low molecular weight, nitrogen‐containing polycations (Asija et al., 2023). In eukaryotes, polyamines are synthesized in a step‐wise process from arginine where the arginine is first converted to ornithine, which is then decarboxylated to putrescine (PUT) via ornithine decarboxylase 1 (ODC1). PUT is then converted via spermidine synthase to spermidine (SPD), which is finally converted to spermine (SPM) via spermine synthase (Cruz‐Pulido & Mounce, 2023). Polyamines play an important role in host‐virus interactions as these are required for transcription, translation, and nucleic acid metabolism (Asija et al., 2023). They are also known to trigger both local and systemic responses against viral infections and alterations in their concentrations have been reported in multiple plants in response to virus attacks (Asija et al., 2023). For example, up to a six‐fold increase in PUT accumulation was observed in phloem sap of *Citrus macrophylla* under citrus tristeza closterovirus (CTV) infection (Nehela & Killiny, 2020). A similar accumulation in spermine concentration has also been reported during the induction of HR in the intercellular spaces of tobacco, which triggers the expression of PR proteins during TMV infection (Yamakawa et al., 1998). In plants, S‐adenosylmethionine decarboxylase (SAMDC) and DNA methyl transferases both frequently use S‐Adenosylmethionine (SAM) as a substrate. Therefore, SAMDC degradation prevents host DNA methylation‐mediated gene silencing, which speeds up viral replication (Moffatt & Weretilnyk, 2001). Intriguingly, a current study found a correlation between a reduction in SAMDC activity during potato virus Y (PVY) infection and the methionine cycle, a key process of the antiviral defence system in plants. Moreover, enhanced spermine and spermidine contents were also reported due to an increase in SPD synthase activity, which in turn results in enhanced defence response against PVY in potato (Glushkevich et al., 2022). PAs, especially SPM, are also associated with the inhibition of virus multiplication by blocking the catalytic activity of hammerhead ribozymes, needed for virus multiplication (Kaddour et al., 2014). A comparative metabolomics study of plants infected by tomato yellow leaf curl virus (TYLCV), showed that the resistant genotype had significantly higher concentrations of the defence compounds feruloylputrescine and castanospermine at 3‐ and 7 days post‐infection, respectively. Additionally, the resistant genotype displayed upregulation of arginine synthase 2 (ARG2) and an increase in Gamma‐aminobutyric acid (GABA) levels, suggesting a critical function for PA metabolism during incompatible plant‐virus interactions (Sade et al., 2015). Contrarily, tomato mottle mosaic virus injection (ToMMV) showed a decrease in PUT, SPM, and SPD concentrations during compatible interactions, pointing that either an increase in PA catabolism leading to the formation of H_2_O_2_, which is necessary to prevent viral replication by inducing PCD, or a change in the biosynthetic pathway leading to the formation of NO. The role of PA catabolism‐mediated formation of H_2_O_2_ and its implication on plant defence was verified in tobacco (Yoda et al., 2003). Tobacco plants infected with TMV show upregulation of genes involved in PA metabolism. In particular, polyamine oxidase breaks down accumulated PA in the apoplast to produce H_2_O_2_, which efficiently induces hypersensitive cell death. PA and ET both share S‐adenosylmethionine (SAM) as a metabolic precursor. Therefore, a reduction in PA levels at the time of viruses‐mediated tissue injury results in an increase in ET synthesis. For example, ET production surges in response to citrus exocortis viroid (CEVd) infection in tomato with concurrent decrease in the PUT levels significantly decreased because of the reduced ornithine decarboxylase (ODC) activity (Nehela & Killiny, 2020; Bellés et al., 1991). In addition to the PA and ET, metabolic crosstalk between PA and jasmonate has also been documented. Exogenous application of MeJA results in an accumulation of conjugated PAs due to increased expression and enzymatic activities of genes associated with the biosynthesis of PAs (Biondi et al., 2003). The MeJA‐induced PAs are also synergistically strengthened by auxins (like IAA), but they are counteracted by N‐benzyladenine, a synthetic cytokinin that seems to work independently of ET (Biondi et al., 2003).

### 3.5 Hydrogen sulfide

H_2_S is an inorganic, water‐soluble, and flammable gas primarily known for its toxic effects and the characteristic smell of rotten eggs. It is a gaseous signalling molecule in plants that has garnered attention due to its emerging role in regulating plant growth, development, and responses to abiotic and biotic stressors (Khan et al., 2022; Choudhary et al., 2022). While its involvement in biotic stress is not extensively documented, there is evidence suggesting its role in plant defence. H_2_S participates in defence signalling by (1) inducing expression of PR and other defence‐related genes, (2) regulating glutathione metabolism, and (3) interacting with other phytohormones like SA, JA, ET, and auxin (Figure 3B) (Choudhary et al., 2022). H_2_S is known to protect against virus‐induced ROS in multiple ways: (1) by reacting and directly neutralizing various ROS (Filipovic et al., 2018), and (2) by activating the antioxidant defence. The direct interactions of H_2_S with biologically relevant oxidants such as ROS, NO, and peroxynitrite are thermodynamically favourable due to their nucleophilic characteristics (de Bont et al., 2022). Although its role as a direct scavenger of ROS is minor, it is more likely that H_2_S is indirectly involved in ROS homeostasis and increases the activity of several enzymes such as glutathione reductase (GR), ascorbate peroxidase (APX) and catalase which are crucial in ROS scavenging (Guo et al., 2015; Chen et al., 2020). *In vitro* assays have shown that *Arabidopsis* cytosolic APX1 can be persulfidated at Cys32 in the presence of H_2_S, which enhances its catalytic activity (Aroca et al., 2015). Additionally, H_2_S is also associated with the inhibition of viral replication by reducing the expression of virus genes encoding for RDR and Coat Protein (CP). In contrast to plants with lower H_2_S levels, those with greater H_2_S levels had increased expression of SA‐dependent PR genes, resulting in improved immune resistance (Pang et al., 2021).

### 3.6 Carrageenans

Sulfated linear polysaccharides known as carrageenans are the main cellular components of red algae‐related seaweeds. Carrageenans are known to exhibit antioxidant activity and are involved in the scavenging of hydrogen peroxide (Sun et al., 2010). It has also been seen that exogenous application of carrageenans elicits defence responses in plants against viruses and viroids. Plants treated with different carrageenans, including λ‐carrageenan and κ/β‐carrageenan exhibit improved resistance against various viruses such as Chlorotic Dwarf Viroid (TCDVd) in tomato (Sangha et al., 2015) and TMV in tobacco (Nagorskaya et al., 2008). The fact that plants treated with carrageenan show a relatively lower abundance of virus and virus transcripts suggests that carrageenans exhibit inhibitory effects on viruses, probably through regulating their replication (Sangha et al., 2015). Moreover, the application of κ/β‐carrageenan also attenuates the virus translocation in *Datura stramonium* by stimulating lytic processes, leading to the destruction of viral particles and preventing their intracellular accumulation (Nagorskaya et al., 2010).

Sulfated fucans, the highly anionic polysaccharides, are the common structural component of marine brown algae and echinoderms. Sulfated fucan oligosaccharides are also shown to induce resistance in tobacco plants to TMV (Klarzynski et al., 2003). Sulfated Polysaccharide 4 (SPS4), isolated from the red alga *Hypnea musciformis*, contains 98% κ‐carrageenan. Notably, exogenous application of SPS4 significantly reduces the TMV infection on tobacco by eliciting the expression of genes related to SA‐dependent defence, including *PR1a*, *PR2*, and *PR5*, along with JA pathway‐dependent genes such as *PR3* and *Def1.2* (Figure 3B) (Ghannam et al., 2013). Another experiment aimed to investigate the antiviral activity of oligosaccharides of κ‐ and κ/β‐carrageenans obtained through different depolymerization methods. The findings showed that the molecular weight of carrageenans has a considerable impact on their antiviral properties and that the high molecular weight carrageenans exhibit greater antiviral activity against TMV infection in tobacco leaves. However, low molecular weight derivatives of κ‐ and κ/β‐carrageenans, generated by mild acid hydrolysis, exhibit higher antiviral activity as compared to the products generated by other methods such as free radical and enzymatic depolymerization, suggesting that the degradation process of polysaccharides affects the antiviral efficacy of carrageenan oligosaccharides in some yet unknown way. Based on these results, it can be hypothesized that either the chemical structure or the molecular weight of oligosaccharides or even both can be a determinative of antiviral action (Kalitnik et al., 2013).

## 4 CONCLUSIONS AND FUTURE PROSPECTS

Viruses are a major threat to sustainable agriculture and global food security. The lack of effective strategies to control these infections has prompted various researchers across the globe to focus on understanding plant‐virus interactions. Naturally resistant plants are a great resource for understanding the mechanisms of viral resistance and, thus, have been extensively studied to identify key components involved in these defence processes. Evidence gathered over the years has highlighted the crucial roles of phytohormones and PGRs in regulating plant defence responses against virus infections (Figure 4). Accumulating data suggest that resistance in otherwise susceptible plants can be induced through the exogenous application or by inducing the activation of one or more phytohormones/PGRs signalling. Besides these endogenous molecules, some other compounds, such as carrageenans, have also been shown to induce defence response in plants by activating the expression of defence genes that function downstream of both SA and JA‐dependent signalling pathways. Notably, an interplay among various phytohormones/PGRs governs the plant responses to invading viral pathogens by limiting their entry and spreading inside the plant tissues and by suppressing viral replication (Figure 4). These significant findings offer valuable insights into developing effective strategies for mitigating viral diseases in crop plants.

However, despite these significant advancements, many questions remain unanswered, such as how varying environmental conditions could affect the synergism and antagonism among diverse phytohormones and PGRs, and what their effects could be on the plant resistance to virus pathogens. Moreover, the fact that early‐phase ABA signalling can change from a defensive role to potentially suppressing immune responses later in the infection process highlights the need to investigate the spatiotemporal dynamics of phytohormones/PGRs interactions during different stages of viral infection. Although the phase‐specific responses of other phytohormones are not well established, a few studies have revealed that JA also plays a phase‐specific response where activation of JA signalling during early stages of virus infection imparts resistance while later phase activation increases susceptibility, most likely due to its antagonistic action on SA (Alberto et al., 2013). Additionally, expanding the studies to other less‐explored PGRs (such as melatonin, BRs, H_2_S, and NO, among others), on which the information is currently limited, may uncover novel regulatory networks that could be targeted to enhance plant immunity. Furthermore, different genetic and molecular tools such as CRISPR‐Cas9 could be employed to dissect and subsequently confirm the specific interactions between hormone receptors, transcription factors like WRKYs, and downstream signalling components to better understand how to fine‐tune the hormonal balance for optimum defence against different viruses and insect vectors.

## AUTHOR CONTRIBUTIONS

Ravi Gupta and Md Salik Noorani conceived the idea and designed the review. The first draft of the manuscript was written by Kritika Shukla, and other authors revised the subsequent drafts of the manuscript. All authors read and approved the final manuscript.

## Data Availability

Data sharing is not applicable to this article as no new data were created or analyzed in this study.
